# Minimally Invasive Sinus Tarsi Approach for Open Reduction and Internal Fixation of Calcaneal Fractures: Complications, Risk Factors, and Outcome Predictors

**DOI:** 10.7759/cureus.21791

**Published:** 2022-01-31

**Authors:** Turki Alajmi, Asmaa F Sharif, Majd A Majoun, Fedaa S Alshehri, Alanood M Albaqami, Mohammed Alshouli

**Affiliations:** 1 Orthopedics and Traumatology, Prince Mohammed Bin Abdulaziz Hospital, Riyadh, SAU; 2 Clinical Medical Sciences Department, College of Medicine, Dar Al Uloom University, Riyadh, SAU; 3 Forensic Medicine and Clinical Toxicology Department, Faculty of Medicine, Tanta University, Tanta, EGY; 4 Medicine and Surgery, Dar Al Uloom University, Riyadh, SAU; 5 Orthopedics (Foot and Ankle), Prince Mohammed Bin Abdulaziz Hospital, Riyadh, SAU

**Keywords:** gissane’s angle, bohler angle, calcaneal fractures, internal fixation, minimally invasive sinus tarsi approach

## Abstract

Open reduction and internal fixation of displaced intraarticular calcaneal fractures remain the gold standard of treatment, but the traditional extensile approach has been associated with relatively frequent complications. The current study aims to evaluate the less invasive sinus tarsi approach and to elaborate on the associated complications, risk factors, and outcome predictors. A retrospective observational study was carried out among 39 patients diagnosed with calcaneal fractures that were operatively treated between January 2019 and January 2020 at a level-one trauma center in Riyadh, Saudi Arabia. Patients were assessed regarding the complications, pre- and postoperative Bohler's angle, Gissane’s angle, calcaneal height, and return to baseline function. Patients older than 60 years show significantly more complications compared to younger patients (p < 0.05). Type IV calcaneal fracture, according to Sander’s classification, showed significantly more complications than other types (p < 0.05). There were significant variations in pre- and postoperative Bohler's angle and calcaneal height (p < 0.05). These variations apply to the Gissane’s angle but do not rise to significant results (p > 0.05). Furthermore, the current study reports a significant moderate direct correlation between delay time and complication incidence (p < 0.05). In conclusion, the minimally invasive sinus tarsi approach has relatively low complications and excellent clinical and radiological outcomes. Older patients and those who are diagnosed with type IV calcaneal factures, besides those presented with more delay, are more associated with unfavorable complications.

## Introduction

Fractures of the calcaneus are the most common tarsal fractures and represent approximately 2% of all fractures; they are almost consistently caused by direct trauma to the foot either due to accidental fall, deliberate suicide attempt, or a motor vehicle collision [1]. Displaced fractures with an intraarticular component represent over two-thirds of calcaneal fractures and, therefore, cause significant functional morbidity [2]. Conservative management of these fractures in whichever form may result in suboptimal functional results and significantly poor patient satisfaction [3]. Therefore, appropriate patient selection for surgical fixation is paramount to achieving satisfactory results in displaced intraarticular calcaneal fractures [4]. Several attempts to establish a universal classification system for calcaneal fractures have been made, but none have drawn ubiquitous acceptance. The first attempt to classify fractures of the calcaneus was made by Malgaiane in 1843; subsequently, Bohler attempted to create the first classification system with prognostic value, followed by the Essex-Lopresti classification system that has outlasted any other classification system [5-7]. Of particular interest to this study is the Sanders classification system, which is relatively easy to implement if computed tomography is available; it also could guide surgical management and provide prognostic value. However, the main drawback of Sanders classification is that it has failed to address the fracture pattern of the anterior process [8].

Classically, the extensile lateral approach to the calcaneus has been utilized to fix intraarticular displaced fractures; an L-shaped skin incision is used with the vertical limb midway between the fibula and the Achilles tendon, while the horizontal limb is in line with the fifth metatarsal [9]. The incision is then carried down to the bone, and using the subperiosteal dissection, a full-thickness fasciocutaneous flap is developed, followed by direct access to the calcaneus and subtalar joint. The primary hazard of this approach is damage to the lateral calcaneal artery that supplies the corner of the flap [10]. Unfortunately, the use of this approach has been associated with many drawbacks including skin breakdown and wound infection [11]. The high percentage of complications following open reduction and internal fixation of calcaneus fractures using lateral extensile exposure led to the development of the less invasive sinus tarsi approach to treat such fractures [12].

The primary aim of this current study was to identify the risk factors and quantitate the rate of complications associated with the sinus tarsi approach; secondary objectives were to display the difference in radiographic measures and their statistical significance and to further correlate them with patient outcomes.

## Materials and methods

Study design and setting

The present study is a retrospective analysis carried out among patients presented to Prince Mohammad bin Abdulaziz Hospital at Riyadh, Saudi Arabia, between January 1, 2019, and January 1, 2020, with a diagnosis of calcaneal fractures. Prince Mohammad bin Abdulaziz Hospital is a 500-bed level-one trauma center located in the eastern province of Riyadh, the capital of Saudi Arabia, and provides secondary healthcare services for the region.

Sampling and sample size

Non-probability convenience sampling was adopted to approach the largest number of patients. The current study enrolled 39 patients diagnosed with calcaneal fractures. The diagnosis was based on the history, clinical examination confirmed by x-ray, and computerized tomography CT scans.

Inclusion criteria

Inclusion criteria were patients diagnosed with closed intraarticular calcaneal fractures with complete medical records and patients who underwent open reduction (as opposed to percutaneous), provided that these patients were aged 16 years and above.

Exclusion criteria

Patients with pre-existing foot and ankle deformities, those with tongue type fractures, or those who managed with surgical approaches other than the minimally invasive sinus tarsi approach were excluded from the current study.

Compliance with ethical standards

Ethical approval was obtained from the corresponding Institutional Review Board at Dar Al Uloom University (approval number: Pro19050002) following the Declaration of Helsinki, 1964, under ID. All patient’s data were handled anonymously to maintain the confidentiality of the patients. Informed consent was waived by the IRB as the data collection was from the medical records.

Data collection

Demographics and Clinical Assessment

All included cases were subjected to thorough history taking, namely, the history of pre-existing comorbidities including but not limited to diabetes, hypertension, peripheral vascular diseases, or previous lower limb fractures. Furthermore, the injury mechanism was reported as either intentional or non-intentional. The demographic data were also reported including age, sex, occupation, and smoking. Initial clinical assessment including Advanced Trauma Life Support® (ATLS®) protocol and vital data measurements followed by a secondary survey and a comprehensive examination of the different body systems was carried out. This was followed by local examination of the foot and ankle with an emphasis on the neurovascular status of the injured extremity. The delay in the timing of surgery between the injury occurrence and the time until definitive fixation was reported for all included cases.

Radiological Studies

Routine radiological studies were conducted including x-rays and CT scans and trauma surveys as indicated. To further increase the internal validity and objectivity of the study, all measurements were reported by blindly two orthopedic surgeons at different times. Based on the finding of CT scans, the fracture was classified according to Sanders’s classification into type I: undisplaced fracture irrespective of the number of fracture lines, type II: two parts fragments or split fractures, type III: three parts fracture or split depression, and type IV: severely comminuted fracture with four or more bone fragments. Other fractures and multisystem trauma were reported as well.

**Figure 1:**
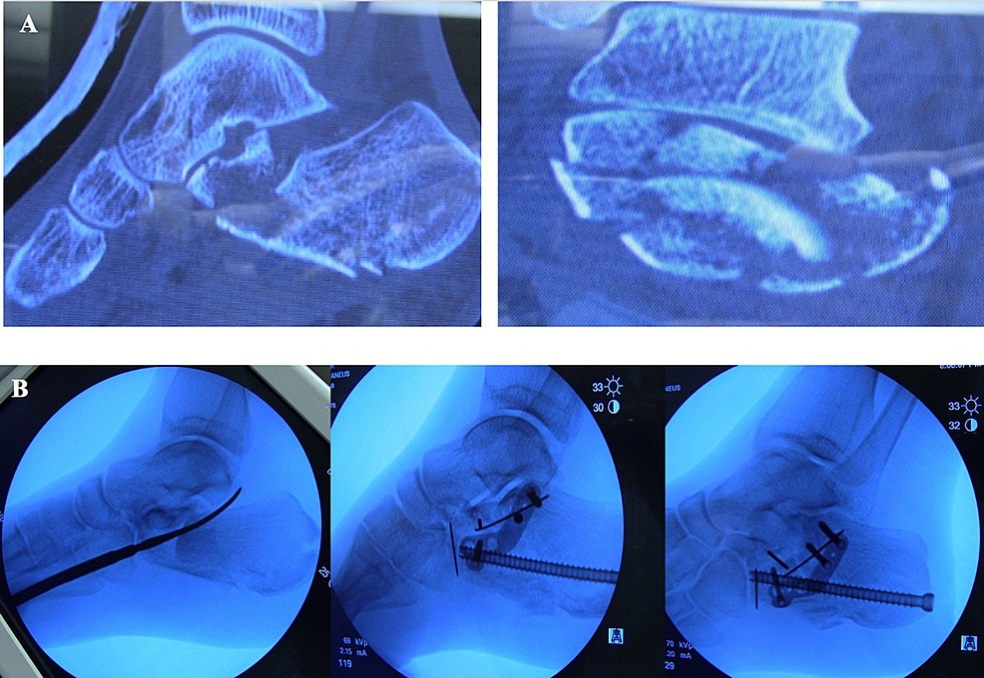
(A) X-rays of calcaneal fractures before surgical approach. (B) X-rays of calcaneal fractures after the minimally invasive sinus tarsi approach

Three measurements were obtained prior to and after fixation. Bohler's angle was measured as the angle outlined by the three landmarks: the apex of posterior tuberosity, the apex of posterior fact, and the apex of anterior process (normal range: 28-40) [13]. Gissane’s angle is the angle extended between the anterior and posterior talar facets of the calcaneus (normal range between 120 and 140) [14]. Also, preoperative and postoperative calcaneal heights were assessed. Figure 1 shows some x-rays obtained before and after conducting the minimally invasive sinus tarsi approach for open reduction and internal fixation of calcaneal fractures.

Surgical technique

**Figure 2:**
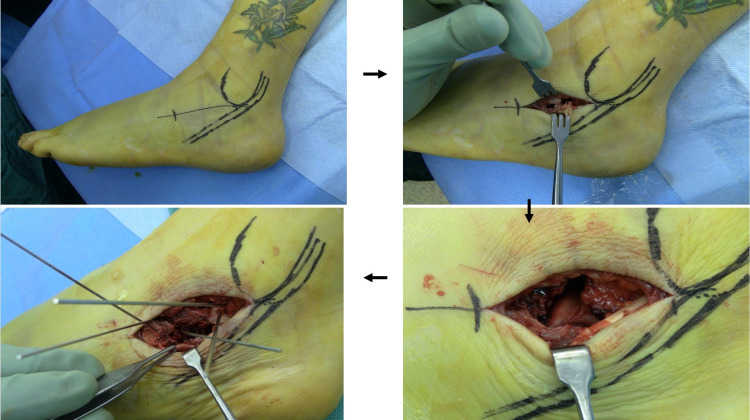
Steps of the minimally invasive sinus tarsi approach for open reduction and internal fixation of calcaneal fractures

The patient was positioned in the lateral position on a regular operating room (OR) table. A bean bag was used to support the patient's body on the table. An image intensifier was used. A centrally threaded Steinmann pin was based through the mid of the calcaneus horizontally confirmed in heel axial views to dis-impact the calcaneus body and correct the valgus varus deformity. A 5-cm horizontal incision was made over the sinus tarsi extending from the base of the fourth metatarsal to the tip of the fibula, lateral side of the foot, mobilization of skin flaps taking care to preserve peroneal tendons and sural nerve, which run at the posterior part of the incision. Then, we separate the outer cortex from the soft tissue using a McDonald’s tool or any available blunt tool [15]. Elevation of the posterior facet and reduction of the articular surface with temporary fixation with K-wires and bone graft were needed. We used two screws of 3.5 mm passed from the distal posterior aspect of the calcaneus directed toward the posterior facet of the calcaneus parallel to each other to maintain the neutral alignment of the heel on its axial views and maintain the height and a posterior to anterior 7.00-mm screw to provide internal support and prevent subarticular collapse. Most of the time, we use the minimally invasive calcaneus plate available from Synthes 2.4/2.7 mm VAL plate through the same approach to achieve more rigid and congruent reduction and fixation. Approximation of the subcutaneous tissue was done using 2/0 vicryl and 3/0 proline vertical knots for the skin. Immobilization on a below-knee cast was used in a neutral position with non-weight bearing on the operated foot [16]. All studied cases were operated by a single surgeon with a certified fellowship in foot and ankle surgery. The sequence of the surgical technique is shown in Figure 2.

Management and follow-up

In addition to standard perioperative preparation, all cases underwent operative repair with the minimally invasive sinus tarsi approach. Routine follow-up was arranged for all patients at varying times according to their socioeconomic status and their area of residence, but all patients were recalled for follow-up six months after discharge. Follow-up of all patients following discharge was conducted by reviewing their clinical data, CT scans, and x-rays. The cases were categorized based on their outcomes into two categories: (1) cases that returned to their baseline function with the ability to commence their previous jobs and activities of daily living, did not need the use of walking aids, and did not use any analgesic medication for their calcaneal fracture; and (2) patients who needed re-admission for complications of the fracture, patients who needed secondary surgery, and patients with associated disabilities that have limited their return to their previous jobs and/or limited their activities of daily living including the use of walking aids and chronic use of analgesia for their calcaneal fractures.

Statistical analysis

Collected data were statistically analyzed using Statistical Package for the Social Sciences (SPSS) software statistical computer package version 26 (SPSS Inc., Chicago, Illinois, USA). ﻿The normality of data distribution was assessed. The data were expressed in numbers and percentages, means, and standard deviations. Paired and unpaired T-tests were used to compare the patients' preoperative and postoperative measurements and to compare the patients of different outcomes. The Chi-square test and independent-sample t-test were used to compare the variables among the patients of different outcomes. The level of significance was 5% with a confidence interval of 95%. The receiver operating characteristic (ROC) curve was plotted to ascertain the effect of delay time as a complication predictor [17].

## Results

Patients’ characteristics

This study enrolled a total of 39 calcaneal fracture patients who were presented to the emergency room (ER) and admitted with a diagnosis of closed calcaneal fractures. The age of included cases ranged between 16 and 62 years with a mean (34.46 + 12.332); 31 males (79.5%) in comparison to eight females (20.5%) were involved. Most of the patients work as manual laborers, 17 (43.6%). Students represented 17.9%, as did unemployed patients; 15.4% were housemaids, while 5.1% worked as drivers. Medical records revealed that 5.1% of the included patients were diabetic, 7.7% hypertensive, and 35.9% smokers. No cases suffering from peripheral vascular diseases were presented (Table 1).

**Table 1:** Demographics and past medical history of patients enrolled in the current study

Variables		Number (out of 39)	Percentage (100%)
Demographics			
Age group	16-<20	4	10.3
	20-<30	7	17.9
	30-<40	16	41.0
	40-<50	8	20.5
	50-<60	2	5.1
	60 and more	2	5.1
Gender	Female	8	20.5
	Male	31	79.5
Occupation	Builder	17	43.6
	Driver	2	5.1
	Student	7	17.9
	Housemaid	6	15.4
	Unemployed	7	17.9
Past medical history			
Diabetes mellitus	No diabetes	37	94.9
	diabetic patient	2	5.1
Hypertension	No hypertension	36	92.3
	Hypertensive patients	3	7.7
Smoking	Non-smoker	25	64.1
	Smoker	14	35.9

Based on the history obtained from the cases, the injuries investigated by the police for probable intentional causes represented 15.4%, while 88.6% of cases were proved unintentional. All the cases investigated for unintentional injuries were housemaids.

Mechanism and types of injuries

**Figure 3:**
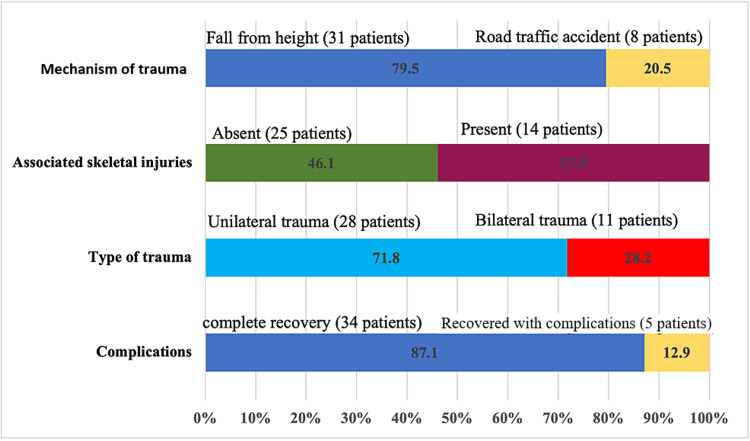
Distribution of mechanism and type of trauma, associated skeletal injuries, and complications among cases diagnosed with calcaneal fracture included in the current study

Figure 3 depicts that 79.5% of the calcaneal fractures were due to a fall from height and 20.5% resulted from road traffic accidents. Unilateral limb trauma represented 71.8% versus 28.2% bilateral lower limb trauma. Association of skeletal injuries was reported in 53.9% and mostly includes skull fractures 56.4%, rib fractures 15.6%, 10% spinal fractures, and 18% upper limb fractures.

**Figure 4:**
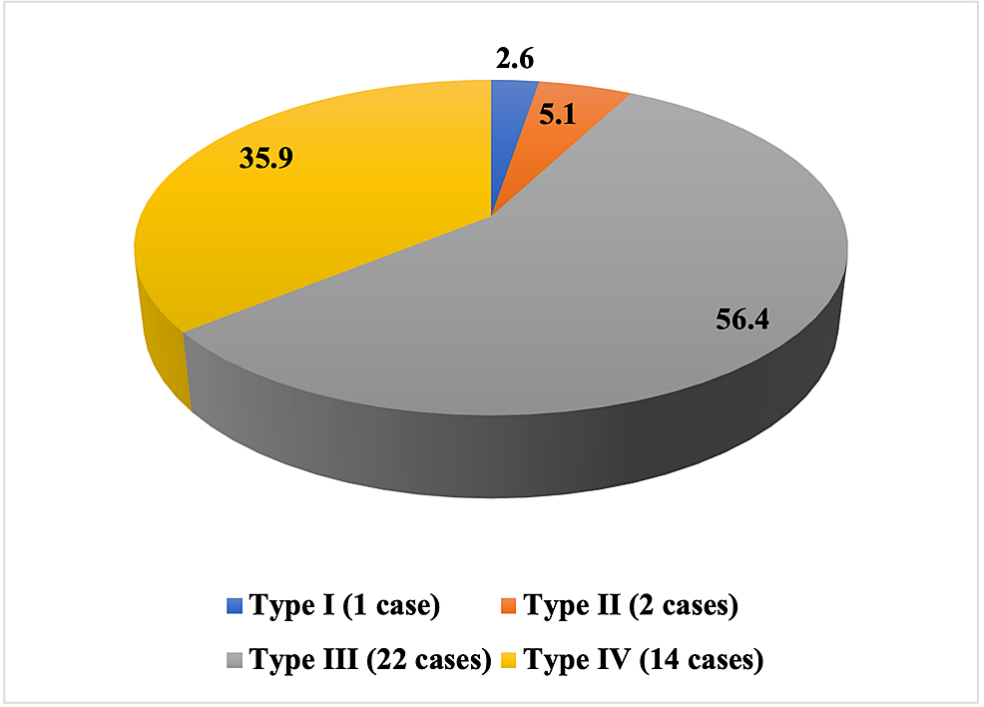
Sander's classification of calcaneal fractures among the studied cohort

The current study reveals that the rate of complications after operative repair with minimally invasive sinus tarsi approach (5/39) * 1000 populations = 128.2. Based on Sander's classification of calcaneal fractures, 2.6% belonged to type I, 5.1% to type II, 56.4% to type III, and 35.9% to type IV (Figure 4). Among the presented cases, complications were reported in 12.9%. Complications were reported in five cases in the form of severe posttraumatic arthritis, malunion, revision to subtalar fusion, and early asymptomatic arthritis. Furthermore, 34 patients (87.1%) returned to their baseline functional status and to their jobs; the remaining five patients (12.9%) required further revision surgery to subtalar arthrodesis within the following six to nine months due to mal-/non-union and early arthritis. There were no cases of deep infection, amputation, or any patients requiring more than one revision surgery. Considering Sander's classification of calcaneal fractures, significantly more complications were encountered in types III and IV than type II (p < 0.05). Furthermore, none of the type I cases encountered significant complications.

Management and complications

Table 2 shows the distribution of the different demographics and fracture criteria among the included cases based on their outcomes. Patients older than 60 years show significantly more complications compared to other groups (p < 0.05). The mean age of patients who developed complications (47.80 + 16.976 years) is significantly higher than the mean age of cases who did not develop complications (32.50 + 10.440), (p < 0.05, 59% CI: -26.295-4.305). Type IV calcaneal fracture according to Sander’s classification showed significantly more complications than other types (c^2^ 8.963, p < 0.05). However, the other demographics, type and mechanism of trauma, and associated skeletal injuries did not show significant variations among the patients of different outcomes.

**Table 2:** Demographics and clinical criteria of the patients enrolled in the current study *Significance < 0.05.

Variables		No complications n (%)	Complications n (%)	Chi-square test	p-values
Age group (years)	16-19	4 (11.8)	0 (0)	*c^2^ *18.470	0.002*
	20-29	6 (17.6)	1 (20)		
	30-39	15 (44.1)	1 (20)		
	40-49	8 (23.5)	0 (0)		
	50-59	1 (2.9)	1 (20)		
	60 and above	0 (0)	2 (40)		
Gender	Female	7 (20.6)	1 (20)	*c^2^* 0.001	0.976
	Male	27 (79.4)	4 (80)		
Occupation	Builder	14 (41.2)	3 (60)	*c^2^* 1.771	0.778
	Driver	2 (5.9)	0 (0)		
	Student	7 (20.6)	0 (0)		
	Housemaid	5 (14.6)	1 (20)		
	Unemployed	6 (17.7)	1 (20)		
Diabetes mellitus	No diabetes	33 (97.1)	4 (80|)	*c^2^* 2.607	0.106
	Diabetic patient	1 (2.9)	1 (20)		
Hypertension	No hypertension	32 (94.1)	4 (80)	*c^2^* 1.224	0.269
	Hypertensive patients	2 (5.9)	1 (20)		
Smoking	Non-smoker	21 (61.8)	4 (80)	*c^2^ *0.630	0.427
	Smoker	13 (38.2)	1 (20)		
Type of trauma	Unilateral	25 (37.5)	3 (60)	*c^2^ *0.934	0.530
	Bilateral	9 (26.5)	2 (40)		
Associated skeletal injuries	No	23 (67.6)	2 (40)	*_c_2* 1.448	0.229
	Yes	11 (32.4)	3 (60)		
Mechanism of trauma	Fall from height	26 (76.5)	5 (100)	*c^2^* 1.480	0.224
	Road traffic accident	8 (23.5)	0 (0)		
Sander’s classification of calcaneal fracture	Type I	1 (2.9)	0 (0)	*c^2^* 8.963	0.03*
	Type II	1 (2.9)	1 (20)		
	Type III	22 (64.7)	0 (0)		
	Type IV	10 (29.4)	4 (80)		

Comparison between radiological measurements by two observers showed no significant variations (p = 0.583). Comparison of preoperative and postoperative measurements of Bohler's and Gissane's angles and calcaneal height among the included patients shows significant variations in the Bohler's angle and calcaneal height (p < 0.05). These variations apply to the Gissane's angle but do not rise to a significant result (p > 0.05) as shown in Table 3.

**Table 3:** Preoperative and postoperative radiological measurements among cases diagnosed with calcaneal fracture according to their outcomes *Significance < 0.05.

	Preoperative	Postoperative	95% CI (lower-upper)	t	P	Paired sample correlation	P-value of correlation
	Mean difference (SD)						
Bohler's angle	-11.051 (9.622)		(-14.170-7.932)	-7.173	<0.001*	0.738	<0.001*
Gissane's angle	3.359 (26.581)		(-5.258-11.976)	0.789	0.435	0.134	0.403
Calcaneal height	-0.4538 (0.4198)		(-0.5899-0.3178|)	-6.751	<0.001*	0.586	<0.001*

However, Table 4 illustrates that the mean of change in Bohler's angle in the group of patients who developed complications is 3.80 (8.044) compared to 12.12 (467) in those who did not develop any complications. Gissane's angle changed by 6.20 (9.859) in the group of patients who developed complications versus -4.76 (28.035) in the group of patients who developed no complications. Changes in calcaneal height were 0.454 (0.420) in all the presented cases.

**Table 4:** Radiological measurements and delay time among the cases diagnosed with calcaneal fracture according to their outcomes *Significance < 0.05. CI, Confidence interval; ORIF, open reduction and internal fixation.

Radiological measurements		All cases	Recovered with no complications	Recovered with complications	Mean difference	95% CI (lower-upper)	Test of significance	p-values
Preoperative Bohler’s angle	Min-max	-38-24	-38-23	0-24				
	Mean (SD)	2.44 (14.033)	1.26 (14.315)	10.40(9.450)	-9.135	-22.5984.327	t-1.375	0.177
Postoperative Bohler’s angle	Min-max	-15-38	-15-38	9-18				
	Mean (SD)	13.49 (12.043)	13.38 (12.861)	14.20 (3.564)	-0.818	-22.598-4.327	t-0.140	0.889
Change in Bohler’s angle	Min-max	-10-40	0-40	-10-10				
	Mean (SD)	11.05 (9.622)	12.12 (467)	3.80 (8.044)	8.318	-6.440-4.804	t-2.108	0.070
Restoration of normal Bohler’s angle	Yes	34	29	5			c^2 ^0.843	0.358
	No	5	5	0				
Preoperative Gissane’s angle	Min-max	38-180	38-180	95-137				
	Mean (SD)	123.59 (25.214)	124.79 (26.294)	115.40 (15.469)	9.394	-15.205-33.994	t-0.774	0.444
Postoperative Gissane’s angle	Min-max	78-145	78-145	115-131				
	Mean (SD)	120.23 (12.575)	120.03 (13.329)	121.60 (5.899)	-1.571	-13.931-10.790	t-0.257	0.798
Change in Gissane’s angle	Min-max	-64-92	-64-92	-6-20				
	Mean (SD)	-3.36 (26.583)	-4.76 (28.035)	6.20 (9.859)	-10.965	-36.851-14.922	t-0.858	0.396
Restoration of normal Gissane’s angle	Yes	16	15	1			c^2 ^1.048	0.306
	No	23	19	4				
Preoperative calcaneal height	Min-max	2.4-4.6	2.4-4.6	3.5-4.6				
	Mean (SD)	3.707 (0.483)	3.771 (0.4908)	3.980 (0.4266)	-0.2094	-0.6794-0.2606	t-0.903	0.372
Postoperative calcaneal height	Min-max	3.5-5.3	3.5- 5	3.7-5.3				
	Mean (SD)	4.251 (0.434)	4.229 (0.4153)	4.400 (0.5788)	-0.1706	-0.5936-0.2525	t-0.817	0.419
Change in calcaneal height	Min-max	-0.4-1.5	0.4-1.5	0.2-0.7				
	Mean (SD)	0.454 (0.420)	0.459 (0.443)	0.420 (0.228)	0.039	-0.374-0.452	t-0.191	0.850
Delay time from admission until ORIF (days)	Min-max	2-55	2-55	14-20				
	Mean (SD)	11.72 (8.805)	10.76 (9.012)	18.20 (2.490)	-7.435	-11.370-3.500	t-3.903	<0.001*

**Figure 5:**
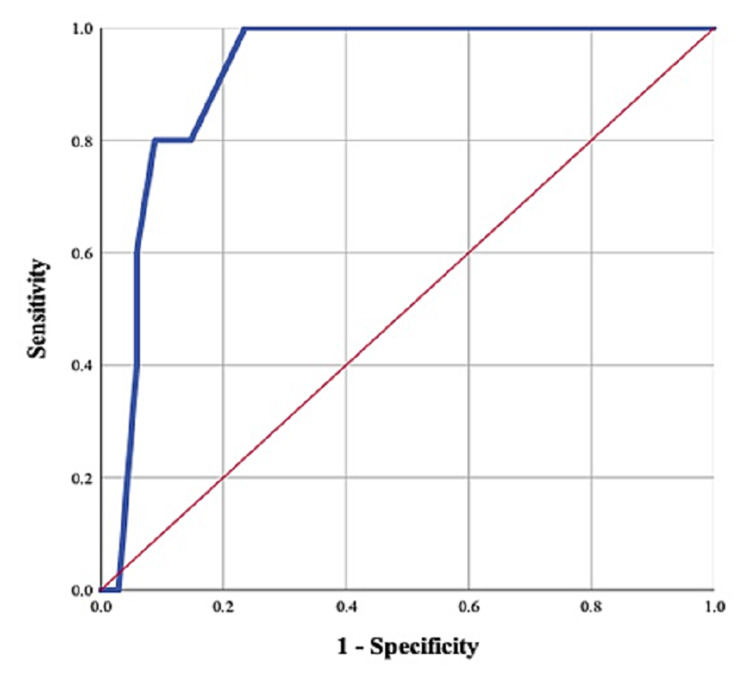
Receiver operating characteristic (ROC) curve of delay time (days) as an outcome predictor among the cases of calcaneal fractures included in the current study

When the delay time from fracture until the operative repair was compared between the patients of different outcomes, significant variations were reported (p < 0.05). Furthermore, the current study reports a significant moderate direct correlation between the delay time and complication incidence (Spearman's correlation coefficient = 0.485, p < 0.05). Figure 5 shows the ROC curve of delay time as an outcome predictor. At cutoff 13.5 days, the delay time is an excellent significant predictor of the incidence of complications (AUC = 0.918, p = 0.003) with sensitivity 100% and specificity 76.5% at 95% CI = 0.286-1.

## Discussion

The first modification of the sinus extensile lateral approach was described by Palmer in 1948, which was an incision curved along the course of the peroneal tendons [18]. However, due to its limited exposure and visibility, it has led to less anatomic reduction and less than satisfactory functional results [19]. Furthermore, several attempts to modify the limited lateral approach has been made, and the most recent sinus tarsi approach has been generalized [20,21].

The most recent and well-described sinus tarsi approach is described as a straight incision centered over the sinus tarsi with minimal soft tissue handling and dissection; using this approach, numerous techniques have been described for exposure and fixation of fractures [16,22].

Several techniques can be used to fix intraarticular calcaneal fractures. Pitts et al. compared plates versus screws in 74 fractures, and there was no difference in the radiographic parameters including Bohler’s angle, Gissane’s angle, and primary reduction at six months follow-up. Furthermore, there was no difference in postoperative complications and operative time [23]. In our current study, both methods were used, and they were dictated by the fracture pattern, extent of comminution, and surgeon’s preference.

Although elderly patients showed an increased rate of complications after undergoing operative treatment, several studies have advocated open reduction and internal fixation to achieve superior outcomes [24,25]. However, important patient selection is invaluable as patients with low functional status, limited ambulation, overwhelming medical comorbidities, and severe osteopenia may be candidates for conservative treatment in selected cases [26].

There is also no general consensus on the optimal timing for calcaneal fractures. In the absence of systemic contraindications to perform open reduction and internal fixation, the delay should not be more than 14 days as soft tissue shrinking and fibrous union will render reduction difficult [27]. On the other hand, if percutaneous reduction and fixation are planned, the delay should be no more than a week to ensure adequate reduction is not obscured by the fracture hematoma and primary stages of fracture healing. Exceptions to these rules include emergencies in the form of open fractures, impending compartment syndrome, and soft tissue incarceration between bony fragments [28].

Furthermore, even with its limited exposure, a large meta-analysis of 1131 patients comparing the minimally invasive sinus tarsi approach with the extensile lateral approach has shown to have a statically significant shorter operative time along with lower complication rates, a smaller number of re-operations, and less postoperative articular displacement [29]. On the other hand, Bai et al. reported no difference in Bohler’s angle, American Orthopedic Foot and Ankle Society score, or the visual analog score, which represent conflicting results to some of the available data. Although the minimally invasive sinus tarsi approach had a shorter operative time, it did not rise to statistical significance [30]. So, our study shows that the minimally invasive sinus tarsi approach represents a safe and efficient alternative to the extensile lateral approach. Furthermore, patient selection and timing to surgery represent critical factors to determine patients' outcomes. To our knowledge, this is a rare and largest study published from Saudi Arabia analyzing the minimally invasive sinus tarsi approach for calcaneal fractures.

Limitations

Although the current evidence shows supporting evidence toward using the minimally invasive approach for fixing calcaneal fractures due to its effectiveness, further large and high-quality randomized control trials are needed to determine the accurate difference between different approaches and subsequent long-term complications. Moreover, comparing the outcomes of the adopted approach with outcomes of calcaneal fracture operated upon by the classic way, plating using extensile approach or conservative management will provide more supporting evidence. However, carrying out the study in one center with a relatively small number of cases is considered the main limitation of the current study. Future multicentered research on larger cohorts is advocated.

## Conclusions

The current study revealed significantly fewer complications and better outcomes among patients diagnosed with calcaneal fractures and managed with the minimally invasive sinus tarsi approach. Complications were reported in the form of severe posttraumatic arthritis, malunion, revision to subtalar fusion, and early asymptomatic arthritis. Most patients returned to their baseline functional status and to their jobs. Complications were encountered in elderly patients aged above 60 years and diagnosed with type IV calcaneal fracture according to Sander’s classification who had been significantly delayed until receiving the surgical and medical therapy.
